# MITOCHONDRIAL-DERIVED PEPTIDE, SHLP2, A NOVEL PROTECTIVE FACTOR IN PARKINSON’S DISEASE

**DOI:** 10.1093/geroni/igz038.3088

**Published:** 2019-11-08

**Authors:** Su Jeong Kim, Anjali Devgan, Hemal H Mehta, Pinchas Cohen

**Affiliations:** Leonard Davis School of Gerontology, University of Southern California, Los Angeles, California, United States

## Abstract

Mitochondrial DNA (mtDNA) variants are associated with a wide range of diseases of aging, from diabetes to Alzheimer’s, as well as with longevity itself. However, to date, little work has thoroughly examined the functional roles of mtDNA variants in such age-related diseases or the therapeutic potential of mitochondrial-derived peptides (MDPs) in these conditions. Our lab hypothesizes that mtDNA SNPs could affect MDPs, and we recently showed that a mtDNA SNP is associated with reduced circulating levels of an MDP called humanin and with cognitive decline. How other mtDNA SNPs affect MDPs and disease risk has yet to be analyzed. Remarkably, a recent paper showed a mtDNA SNP (m.2158 T>C) reduces the risk of Parkinson’s disease (PD). Of note, this SNP changes lysine (K) 4 to arginine (R) of a MDP called SHLP2, which is encoded by the 16S rRNA region of the mtDNA. SHLP2 acts as a neuroprotective factor and as a metabolic regulator. We hypothesized that K4R SHLP2 – produced by individuals who carry mtDNA m.2158 T>C – is a protective factor for Parkinson’s disease. Cycloheximide-treated pulse-chase experiments additionally showed that K4R SHLP2 is more stable than WT SHLP2. WT SHLP2 has a polyubiquitination whereas K4R SHLP2 diminish the polyubiquitination. K4R SHLP2 more potently inhibits PD toxin (MPP+) induced apoptosis in neuronal cells. K4R SHLP2 reverse the mitochondrial membrane potential loss and mitochondria respiration defect in TFAM heterozygous knockout MEFs. Altogether, SHLP2 has the therapeutic potential as a precision medicine in PD.

